# How to avoid unnecessary surgical treatment for neonatal small left colon syndrome

**DOI:** 10.1093/jscr/rjab072

**Published:** 2021-04-19

**Authors:** Taro Ikeda, Shumpei Goto, Takashi Hosokawa, Toshiki Rikiyama, Shigeharu Hosono, Kenjiro Takagi

**Affiliations:** Department of Surgery, Saitama Medical Center, Jichi Medical University, Saitama, Japan; Department of Surgery, Saitama Medical Center, Jichi Medical University, Saitama, Japan; Department of Surgery, Saitama Medical Center, Jichi Medical University, Saitama, Japan; Department of Surgery, Saitama Medical Center, Jichi Medical University, Saitama, Japan; Department of Perinatal Medicine, Saitama Medical Center, Jichi Medical University, Saitama, Japan; Department of Perinatal Medicine, Saitama Medical Center, Jichi Medical University, Saitama, Japan

**Keywords:** functional gastrointestinal obstruction, gestational diabetes, Hirschsprung’s disease, neonate

## Abstract

We report a case of neonatal small left colon syndrome (NSLCS) that underwent surgery. A female infant was born at 38 weeks of gestation. The mother had gestational diabetes requiring insulin therapy. The baby was admitted for respiratory distress. Abdominal distension was observed, and the gastric residue increased. Contrast enema revealed a small caliber of the left colon up to the splenic flexure. At 14 days, the full-thickness biopsy of the sigmoid and transverse colons was performed. Pathological diagnosis showed that the sigmoid colon had few ganglion cells, therefore the transverse colostomy was performed. At 6 months of age, a rectal biopsy was performed to confirm the diagnosis of Hirschsprung’s disease; the intestinal plexus and ganglion cells were normal. The surgery was changed from a pull-through to a stoma closure. The postoperative diagnosis was NSLCS, and the course up to 3 years was good without defecation or growth problems.

## INTRODUCTION

Neonatal small left colon syndrome (NSLCS), originally reported in 1974 by Davis *et al* [1], is a rare disease involving gastrointestinal functional obstruction. In Japan, NSLCS is extremely rare, with only a few cases since the first report in 1983 [2]. NSLCS develops in association with gestational diabetes and it often recovers spontaneously. We operated on an infant with NSLCS and discuss how surgery could potentially be avoided.

## CASE REPORT

**Figure 1:**
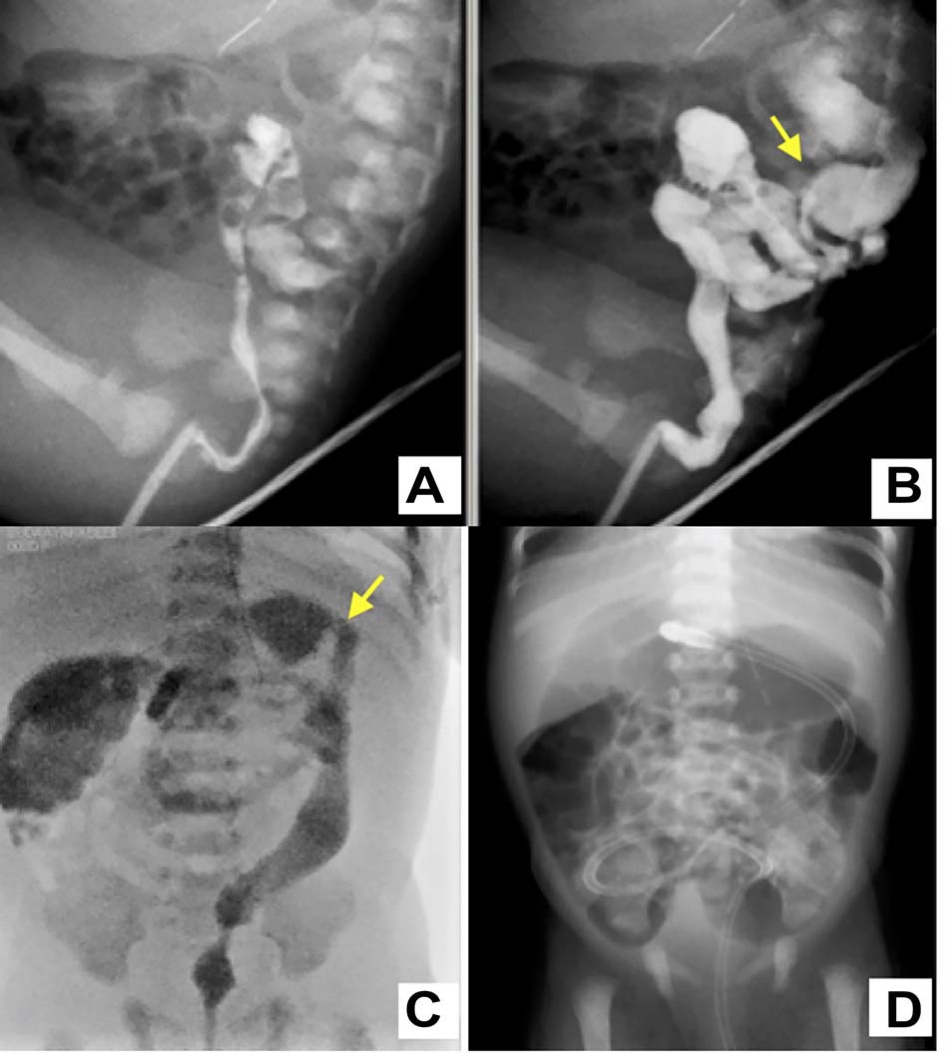
A water-soluble contrast enema image shows characteristic narrowing of the left colon up to the splenic flexure (yellow arrow) (**A**–**C**); a transanal tube was inserted into the transverse colon (**C**); the plain abdominal X-ray of the occluded tube shows dilation of the colon from the transverse colon to the oral side (D).

A female infant was born at 38 weeks of gestation by cesarean section. Birth weight was 3002 g and height was 49 cm. During antenatal period, the mother had gestational diabetes requiring insulin therapy from 12 weeks of gestation. The postnatal course was good, insulin treatment was discontinued and she was discharged 6 days after delivery. The baby was admitted to the neonatal unit for respiratory distress, which required respiratory management with directional positive airway pressure. Abdominal distension was observed from 8 h after birth, and the amount of milk-like gastric residue increased. The passage of meconium was confirmed by glycerin enema, but abdominal distension persisted. An abdominal X-ray showed dilated bowel loops and absent rectal gas. Therefore, intermittent continuous gastric suction was started. Contrast enema revealed a small caliber of the left colon up to the splenic flexure at 3 days of age (Fig. 1A–C.). A transanal tube was inserted into the transverse colon to initiate intestinal decompression. Subsequently, the abdominal distension was improved, and the amount of feeding could increase; intermittent intragastric continuous suction was ceased at 7 days. The next day, she had a recurrence of abdominal distension with accidental occlusion of the transanal tube (Fig. 1D). Although the symptom was improved by releasing the tube blockage, colostomy was performed because tube management at home could cause the same issue.

**Figure 2:**
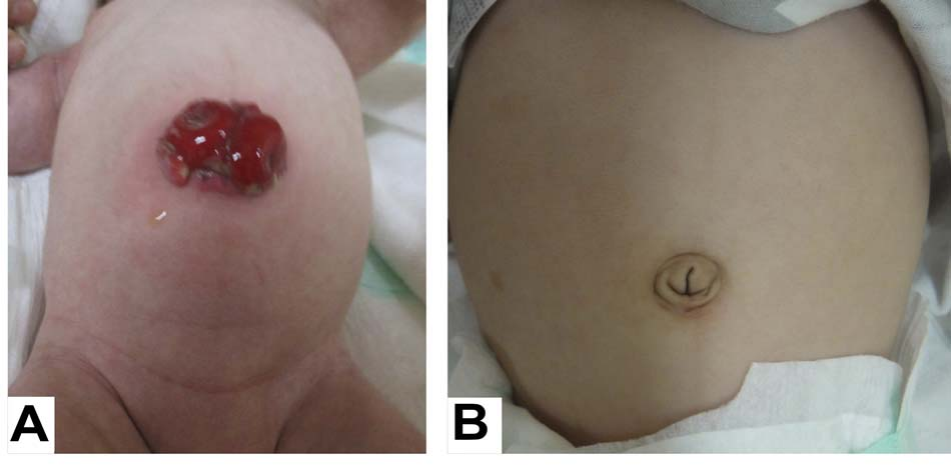
A looped transverse colon stoma was placed in the umbilicus (**A**); the stoma scar on nabel is inconspicuous (**B**).

At 14 days of age, surgery was performed using the umbilicus approach, and first, a full-thickness biopsy of the sigmoid and transverse colons was performed. Pathological diagnosis showed that the sigmoid colon had few intestinal ganglion cells and the transverse colon was normal, so a transverse colon stoma was placed in the umbilicus (Fig. 2A). Pathological findings suggest that the sigmoid colon is part of a long transition zone in Hirschsprung’s disease (HD). While waiting for weight gain, we decided to perform a pull-through for HD. First, a rectal biopsy was performed to confirm the diagnosis of HD. As a result, the intestinal plexus and ganglion cells were diagnosed as normal. Therefore, the operation was changed from a pull-through to a stoma closure. The postoperative diagnosis was NSLCS based on the clinical course and the findings of enema examination after reviewing the previous and current pathologies. The 3-year follow-up revealed a good postoperative course without scar or defecation or growth problems (Fig. 2B).

## DISCUSSION

NSLCS is one of the functional intestinal obstructions that develop in newborns; it is characterized by a narrow left colon with an abrupt transition zone at the splenic flexure on contrast enema. Meconium plug syndrome, meconium ileus and HD are clinically similar but are distinguished by the absence of meconium retention and the lack of ganglion cells in the distal intestinal tract. The etiology is unknown, but there are various theories such as functional immaturity of the ganglion cells, abnormalities of the autonomic nervous system and drug use by the mother. In particular, there is a significant association between gestational diabetes and NSLCS [1, 3]. It was also reported that 1.5% of newborns with constipation and abdominal distension had NSLCS, and it has been proposed that the NLSCS prevalence may be higher than previously reported [4]. NSLCS often improves spontaneously, with improvement in 1–2 weeks.

In this case, there was abdominal distension from the neonatal period, and HD was suspected by contrast enema. Although the symptoms were improved by transanal tube drainage, a stoma was constructed due to tube trouble. It was possible that colostomy could have been avoided if reassessed during the 1-week waiting period before surgery. A rectal biopsy just prior to the pull-through confirmed normoganglia, and further intervention was avoided. Therefore, rectal biopsy should be performed first when performing surgical treatment, including colostomy.

In neonatal intestinal obstruction, it is important to recognize NSLCS as a disease to be differentiated, especially in the case of maternal diabetes. However, it is difficult to differentiate between HD and NSLCS in infants with a caliber change. Conservative treatment should be considered for at least 2 weeks if symptoms do not improve, and a rectal biopsy should be performed before surgical treatment,, including colostomy.

## CONFLICT OF INTEREST STATEMENT

None declared.

## FUNDING

None.
